# Laser-Induced Ablation of Hemp Seed-Derived Biomaterials for Transdermal Drug Delivery

**DOI:** 10.3390/ijms26167852

**Published:** 2025-08-14

**Authors:** Alexandru Cocean, Georgiana Cocean, Silvia Garofalide, Nicanor Cimpoesu, Daniel Alexa, Iuliana Cocean, Silviu Gurlui

**Affiliations:** 1 Laboratory of Applied Meteorology and Climatology, RECENT AIR, Research Center with Integrated Techniques for Atmospheric Aerosol Investigation in Romania, Institute of Interdisciplinary Research, Alexandru Ioan Cuza University of Iasi, A Building, Physics, 11 Carol I, 700506 Iasi, Romania; alexcocean@yahoo.com (A.C.); silvia.garofalide90@gmail.com (S.G.); 2 Atmosphere Optics, Spectroscopy and Laser Laboratory (LOASL), Faculty of Physics, Alexandru Ioan Cuza University of Iasi, 11 Carol I Bld., 700506 Iasi, Romania; cocean.georgiana@yahoo.com (G.C.); nicanor.cimpoesu@tuiasi.ro (N.C.); 3 Rehabilitation Hospital Borsa, 1 Floare de Colt Street, 435200 Borsa, Romania; 4 Faculty of Material Science and Engineering, Gheorghe Asachi Technical University of Iasi, 59A Mangeron Bld., 700050 Iasi, Romania; 5 Faculty of General Medicine, Grigore T. Popa University of Medicine and Pharmacy of Iasi, 16 Universității Street, 700115 Iasi, Romania; alexadaniel2004@yahoo.com; 6 Neurology Medical Rehabilitation Clinical Department, Clinical Rehabilitation Hospital of Iasi, 14 Pantelimon Halipa Street, 700661 Iasi, Romania

**Keywords:** transdermal drug delivery systems, cannabinoids, phenolic acids, endocanabinoid system (ECS), artificial biocomposites, dual pulsed laser

## Abstract

Numerous studies on specific cannabis compounds (cannabinoids and phenolic acids) have demonstrated their therapeutic potential, with their administration methods remaining a key research focus. Transdermal drug delivery (TDD) systems are gaining attention due to their advantages, such as painless administration, controlled release, direct absorption into the bloodstream, and its ability to bypass hepatic metabolism. The thin films obtained via pulsed laser deposition consist of micro- and nanoparticles capable of migrating through skin pores upon contact. This study investigates the interaction of phenolic compounds in hemp seeds with pulsed laser beams. The main goal is to achieve the ablation and deposition of these compounds as thin films suitable for TDD applications. The other key objective is optimizing laser energy to enhance the industrial feasibility of this method. Thin layers were deposited on glass and hemp fabric using dual pulsed laser (DPL) ablation on a compressed hemp seed target held in a stainless steel ring. The target was irradiated for 30 min with two synchronized pulsed laser beams, each with parameters of 30 mJ, 532 nm, pulse width of 10 ns, and a repetition rate of 10 Hz. Each beam had an angle of incidence with the target surface of 45°, and the angle between the two beams was also 45°. To improve laser absorption, two approaches were used: (1) HS-DPL/glass and HS-DPL/hemp fabric, in which a portion of the stainless steel ring was included in the irradiated area, and (2) HST-DPL/glass and HST-DPL/hemp fabric—hemp seeds were mixed with turmeric powder, which is known to improve laser interaction and biocompatibility. The FTIR and Micro-FTIR spectroscopy (ATR) performed on thin films compared to the target material confirmed the presence of hemp-derived phenolic compounds, including tetrahydrocannabinol (THC), cannabidiol (CBD), ferulic acid, and coumaric acid, along with other functional groups such as amides. The ATR spectra have been validated against Gaussian 6 numerical simulations. Scanning electron microscopy (SEM) and substance transfer tests revealed the microgranular structure of thin films. Through the analyzes carried out, the following were highlighted: spherical structures (0.3–2 μm) for HS-DPL/glass, HS-DPL/hemp fabric, HST-DPL/glass, and HST-DPL/hemp fabric; larger spherical structures (8–13 μm) for HS-DPL/glass and HST-DPL/glass; angular, amorphous-like structures (~3.5 μm) for HS-DPL/glass; and crystalline-like structures (0.6–1.3 μm) for HST-DPL/glass. Microparticle transfer from thin films on the hemp fabric to the filter paper at a human body temperature (37 °C) confirmed their suitability for TDD applications, aligning with the “whole plant medicine” or “entourage effect” concept. Granular, composite, thin films were successfully developed, capable of releasing microparticles upon contact with a surface whose temperature is 37 °C, specific to the human body. Each of the microparticles in the thin films obtained with the DPL technique contains phenolic compounds (cannabinoids and phenolic acids) comparable to those in hemp seeds, effectively acting as “microseeds.” The obtained films are viable for TDD applications, while the DPL technique ensures industrial scalability due to its low laser energy requirements.

## 1. Introduction

We have previously studied and reported the high-power pulsed laser interaction with organic molecules, including polymers, contained in various natural biocomposites, such as wool fibers, horns, turmeric, oyster shells, and hemp stalks, resulting in thin films of similar and even identical components as in the precursor material used as an ablation target, aiming to develop a new concept of transdermal drug delivery (TDD) system construction [1,2,3,4,5]. To make the process more accessible to wider applications, including at an industrial level, a lower pulsed laser energy must be used. The feasibility of integrating this technology into commercially available products may pave the way for new transdermal systems. Continuing our studies, in order to reduce the laser energy while maintaining the effect, a dual pulse laser (DPL) system was developed, and thus thin films of chitosan with crystalline structures were obtained from oyster shells [3].

On the same topic, we present, through this paper, the results of the study of the interaction of DPL with the natural biocomposite of hemp seeds, focusing in particular on the behavior of phenolic compounds during ablation and deposition on various supports. Hemp seeds are an alternative as a study material, being a resource of components, such as oils, phenolic compounds, proteins, and fibers, and of great interest for applications in several sectors, including and even predominantly pharmaceutical. Among the phenolic compounds contained in hemp seeds and other parts of the cannabis plant (flowers and leaves), of increasing interest are cannabinoids and especially tetrahydrocannabinol (THC) and cannabinidol (CBD). It is estimated that the number of cannabinoids is more than 100, but few have been identified, and of those identified not all are pharmacologically active [6,7]. The composition of hemp seeds has been intensively studied [8,9,10]. Following the effects of THC on the brain, the researchers discovered two G protein-coupled cannabinoid receptors, named as CR1 and CR2, corresponding to THC and CBD, respectively [6,11,12,13,14,15,16]. The existence of these receptors in mammals induced the idea that their body would produce similar compounds, and thus endocannabinoids were discovered, the first being anandamide [13,17].

The chemical structure of endocannabinoids is amide, unlike main plant cannabinoids (also called phytocannabinoids). Researchers have found amide compounds, similar to anandamide, called lignanamides in hemp seeds [18,19]. Studies on endocannabinoid systems have shown that endocannabinoids are not produced all the time but only in certain conditions. Endocannabinoids bind to receptors and generate reactions that lead to the transmission or not of some substances and to various changes. The same system works in phytocannabinoids [13]. Once endocannabinoid systems were discovered, the action of phytocannabinoids on the human body began to be viewed differently, and CBD and THC have been intensively studied, finding a number of benefits for medicine. They are used as an appetite stimulant agent in anorexia, cancer, or immunodeficiency virus infection and acquired immunodeficiency syndrome (HIV/AIDS) and they also have beneficial antiemetic effects for cancer chemotherapy patients, and provides pain relief for cancer patients, patients with HIV/AIDS, and in the case of other types of chronic pain, such as patients with fibromyalgia and rheumatoid arthritis and multiple sclerosis [20]. Cannabinoid receptors have been identified mainly in the brain and also in other parts of the body such as those in parts of the anterior eye [21].

Since 1985, the Food and Drug Administration (FDA) has approved the marketing of two cannabinoids derived from Δ9-THC, namely dronabinol and nabilone. These two pharmaceutical compounds are used to treat chemotherapy-induced nausea and vomiting, including in patients with anorexia or in patients with acquired immunodeficiency syndrome (AIDS) [22].

A promising prospect is the use of CBD and THC in relieving the symptoms in multiple sclerosis (MS), such as the effects on urinary symptoms, but also on the immune system [23,24]. Preclinical studies have highlighted the role of cannabinoids, including CBD, in the myelination process in diseases such as MS, stroke, and traumatic brain injury (TBI) [22,25]. Research is currently underway into the antioxidant effects of CBD on oligodendrocyte progenitor cells (OPCs) from which the oligodendrocytes responsible for producing mature myelin are derived. The mechanism is independent of CB1 and CB2 receptors, and cells treated with CBD show less oxidative stress [22].

Researchers believe that an important role in modulating the action of cannabinoids is also played by terpenes and the presence of cannabinoid species together [14,20]. Thus, CBD reduces the harmful psychoactive effects of THC, while retaining its pharmacological benefits [20]. Other phenolic compounds in hemp seeds are phenolic acids (coumaric and ferulic) which are already used in the pharmaceutical and cosmetic industries due to their curative effects on the skin [4]. The synergistic action of the chemical compounds in hemp seed makes the use of the whole more interesting than the separation by components. As a whole, hemp seeds have been shown to have antimicrobial effects, [26] with studies showing efficacy against Bacillus cereus, Listeria monocytogenes, and Enterococcus faecalis, and antifungal activity to a lesser extent [27]. The method of administration is currently summarized as only orally in the form of gelling capsules for the two synthetic cannabinoid derivatives imitating the THC molecule, namely dronabinol and nabilone, while CBD is increasingly used as an adjunct in dietary supplements or in various pharmaceutical products. The researchers also investigated other cannabinoid delivery possibilities such as intranasal, pulmonary, oromucosal, and transdermal. Colloids with hemp extracts have also been prepared in an attempt to streamline the administration of cannabinoids that orally lose their properties due to their chemical instability and rapid metabolism [28]. The interaction of cannabinoids with the endocannabinoid system in the skin also represents a potential treatment for certain dermatological conditions, with studies being in the preclinical stage [29,30]. Promising results were obtained in the study of the effects of transdermal patches based on cannabidiol (CBD) in the treatment of psoriasis, including a reduction in itching through action on the CB1, CB2, and TRP channels located in skin nerve fibers, mast cells, and keratinocytes [31].

Transdermal drug delivery (TDD) systems are medical devices that ensure the transfer of the active substance through the skin and its passage into the bloodstream. Among these devices, an important category is transdermal patches. These can be of complex structures (external support, rate controlling membrane, plasticizer, penetration activators, active substance, reservoir, and polymer matrix) or simplified when the active substance is embedded in a polymer matrix or in the adhesive layer [32].

Transdermal patches represent a growing field of drug delivery systems, offering unique advantages in medication administration, patient compliance, and therapeutic outcomes. Our ongoing studies are exploring regulatory pathways for dual pulse laser systems. The purpose of the present study is to investigate the technological effects of the dual pulse laser mechanism on the phenolic components of the natural biocomposite represented by the hemp seed, aiming at the development of transdermal materials capable of delivering their components through skin contact and thus leading to new advanced therapeutic methods. Obtaining thin film structures that can release microparticles under certain temperature conditions or under other stimuli represents an additional advantage over the TDD systems developed to date. Since each microparticle released from the thin layer contains a complex of active substances with a synergistic effect, as well as emollients (oils) and other compounds with a role in ensuring the transdermal passage, the thin films of which they are part of can be considered smart materials.

Using hemp-derived materials aligns with the growing demand for sustainable and eco-friendly medical solutions. The dual pulsed laser effect on phenolic components in hemp seeds presents a promising avenue for developing advanced materials for transdermal drug delivery systems. The natural origin of these phenolic compounds reduces the reliance on synthetic polymers leading to safer and biodegradable options for drug delivery systems.

**Figure 1:**
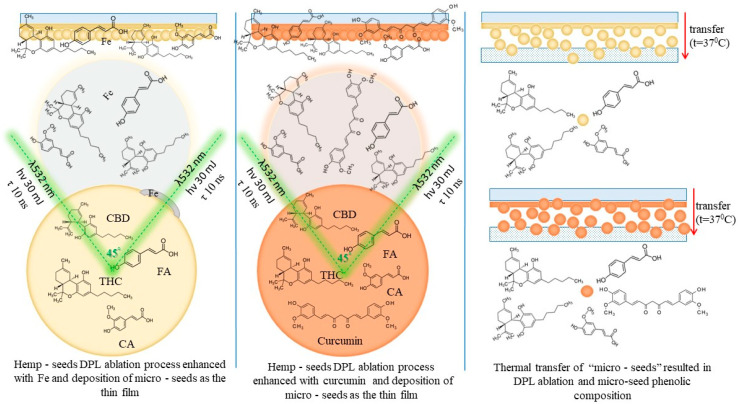
Experimental setup for thin film deposition using the dual pulsed laser technique applied to hemp seeds.

The experimental procedure is schematically presented in Figure 1.

## 2. Results and Discussions

**Figure 2:**
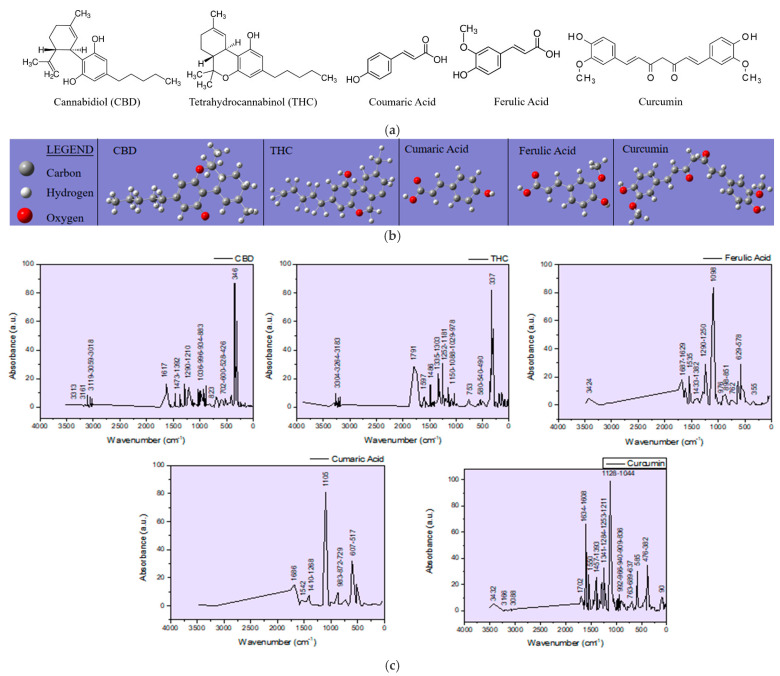
CBD, THC, Ferulic Acid, Coumaric Acid, and Curcumin 2D (**a**) and 3D (**b**) structural chemical formulas and their IR spectra obtained by Gaussian simulation (**c**).

In this study, the main observations are focused on phenolic compounds (namely the CBD and THC cannabinoids and the ferulic and coumaric phenolic acids) and how they are affected during the ablation process and deposition in the thin films induced in the DPL mode (Figure S2, Tables S3 and S4). Curcumin which was added for enhancing the hemp seed ablation is also included in the observations due to its phenolic functional group that may interfere with the targeted compounds in the analysis. Structural chemical formulas of the molecules, including the cannabinoids, CBD and THC, the phenolic acids, coumaric and ferulic, and curcumin, and their IR spectra, were simulated (Figure 2) with the GAUSSIAN 6 software in order to distinguish the vibration bands in the FTIR spectra of the targets and of the thin films specific to hemp and turmeric, which depend on the functional groups in the molecules of the phenolic compounds. The hemp seed target composition was analyzed before and after ablation (HS-target and HS-target ablated), and the spectra proved to be similar. Although the laser beams also hit the stainless steel ring, the eventual re-deposition of the removed material did not produce any observable changes. Also, the interaction of the laser radiation with the hemp seeds did not induce any noticeable modification in the IR spectrum of the ablated target.

**Figure 3:**
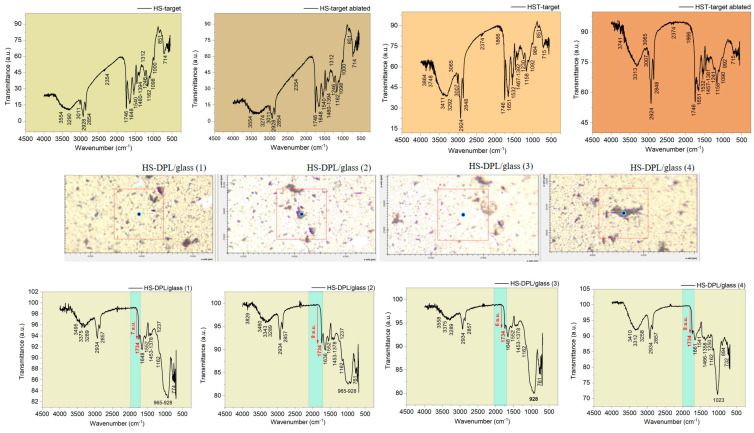

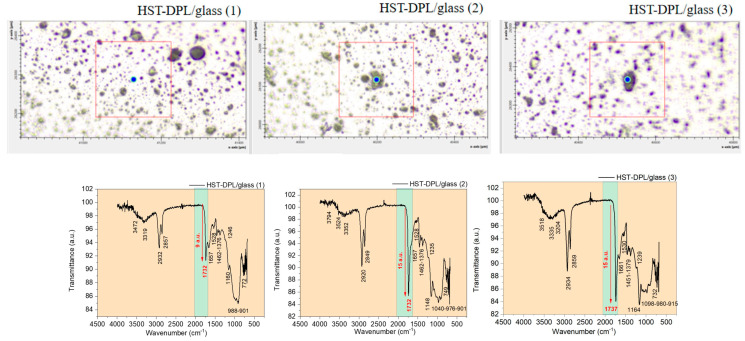
FTIR spectra of initial hemp seed target (HS-target) and the ablated area (HS-target ablated); Micro-FTIR ATR images and spectra of the obtained thin film on the glass slab (HS-DPL/glass (1), HS-DPL/glass (2), HS-DPL/glass (3), and HS-DPL/glass (4)) and of the initial target of hemp seeds mixed with turmeric (HST-target) and the ablated area (HS-T-target ablated); Micro-FTIR ATR images and spectra of the obtained thin film on the glass slab (HST-DPL/glass (1), HST-DPL/glass (2), and HST-DPL/glass (3)).

Regarding the spectra obtained for the deposited thin films on different areas, including HS-DPL/glass (1), HS-DPL/glass (2), HS-DPL/glass (3), and HS-DPL/glass (4) as seen in Figure 3, the vibration bands have remained similar to those of the target (HS-target and ablated HS-target), and only slight shifts in the immediate vicinity of the wavenumber values were recorded. Also, the intensity of the transmittance changed, probably due to the amount of material, but also due to the new arrangement and distribution of its components in the analyzed samples of thin films, as well as due to the analysis method used (transmittance on pellets versus ATR on a thin layer). On the thin layer deposited on the glass support, it is found that there are no major differences between the light color structures (predominant) and the dark ones. Again, only the quantity of components may be different.

Phenolic compounds are highlighted in the FTIR spectra of the ablated HS-target/HS-target and the ablated HS-T target/HS-T target (Figure 3) by the large stretching band in the range of 3554 cm^−1^–3290 cm^−1^/3274 cm^−1^ and 3411–3292 cm^−1^/3313 cm^−1^, respectively, and the bends in the range of 1394–1312 cm^−1^ and 1392 cm^−1^/1381 cm^−1^, respectively, which are assigned to the OH groups of phenols. In the literature [33,34], the aromatic vibrations CH are also assigned in the range from 3200 cm^−1^ to 3312 cm^−1^ and as the bands at 1246 cm^−1^ and 1162 cm^−1^ in the HS target spectra, and 1230 cm^−1^/1241 cm^−1^ and 1158 cm^−1^ in the HS-T target spectra are for Ar-C-OH bindings, and the phenols are once again confirmed. The HS-DPL/glass thin films spectra (Figure 3) show vibrations in the same range as the HS target (HS-DPL/glass (1): 3495 cm^−1^, 3375 cm^−1^, and 3289 cm^−1^ with the bindings at 1378 cm^−1^, 1237 cm^−1^, and 1162 cm^−1^; HS-DPL/glass (2): 3460 cm^−1^, 3343 cm^−1^, and 3289 cm^−1^ with 1378 cm^−1^, 1237 cm^−1^, and 1162 cm^−1^; HS-DPL/glass (3): 3558 cm^−1^, 3375 cm^−1^, and 3289 cm^−1^ with 1378 cm^−1^ and 1162 cm^−1^; HS-DPL/glass (4): 3410 cm^−1^, 3312 cm^−1^, and 3258 cm^−1^ with 1358 cm^−1^, 1250 cm^−1^, and 1162 cm^−1^). In the HST-DPL/glass thin film spectra (Figure 3), slight differences are noticed compared to the HS-T target (HST-DPL/glass (1): 3472 cm^−1^ and 3319 cm^−1^ with bends at 1376 cm^−1^, 1246 cm^−1^, and 1160 cm^−1^; HST-DPL/glass (2): 3524 cm^−1^ and 3352 cm^−1^ with 1376 cm^−1^, 1235 cm^−1^, and 1148 cm^−1^; HST-DPL/glass (3): 3518 cm^−1^ and 3335 cm^−1^ with 1379 cm^−1^, 1239 cm^−1^, and 1164 cm^−1^). The shifts from 3411 cm^−1^ to 3472 cm^−1^, 3524 cm^−1^, and 3518 cm^−1^ in the HST-DPL/glass spectra compared to HS-T target spectrum could be due to the increased deposition of coumaric acid and curcumin, based on the Gaussian simulated IR spectra (Figure 2 and Table S1). In the HS-T target spectrum, there are also stretching vibrations at 3855 and 3748 cm^−1^ assigned to free OH in the silanol terminal groups from turmeric [4]. The C=O functional groups vibrate at 1746 cm^−1^, 1734 cm^−1^, 1746 cm^−1^, 1732 cm^−1^, 1737 cm^−1^ and 1648 cm^−1^, 1636 cm^−1^, 1661 cm^−1^, 1651 cm^−1^, 1657 cm^−1^, and 1661 cm^−1^ in carboxylic acids (ferulic and coumaric acids) when coupled with the bands at 1460 cm^−1^, 1453 cm^−1^, 1466 cm^−1^, 1467 cm^−1^, 1457 cm^−1^, 1462 cm^−1^, and 1451 cm^−1^. The latter bands are also assigned to the methyl and methylene group in curcumin. Aliphatic C-H in the side chains of the studied components are evidenced by the asymmetric and symmetric stretches at (1) 2928 cm^−1^, 2934 cm^−1^, 2924 cm^−1^, 2932 cm^−1^, 2920 cm^−1^ and (2) 2854 cm^−1^, 2857 cm^−1^, and 2848 cm^−1^, respectively. The unsaturated side chain specific to canabinoids and also to coumaric and ferulic acids is shown by specific C=C bends at 1540 cm^−1^, 1552 cm^−1^, 1541 cm^−1^, 1532 cm^−1^, 1528 cm^−1^, and 1530 cm^−1^ coupled with the bends in the range of 1100 cm^−1^ to 700 cm^−1^. Aminoacids, amides, esters, and other components specific to the hemp seeds are also indicated by the vibrations in the IR spectra (Figure 3) as presented in Table S1. Lignanamides (named as cannabisin) [18] may also be indicated by the functional group vibrations presented in Table S1.

Based on the theoretical IR spectra generated with Gaussian software, phenolic compounds can be distinguished in the natural biocomposite structures represented by hemp seeds, as well as in the artificial biocomposites obtained by DPL ablation and the deposition process. Thus, the cannabinoids CBD and THC have specific vibrations at (1) 3313 cm^−1^, 3161 cm^−1^, 3119 cm^−1^, 3059 cm^−1^, 3018 cm^−1^, 1617 cm^−1^, 1473 cm^−1^, 1392 cm^−1^, 1290 cm^−1^, 1036 cm^−1^, 966 cm^−1^, 934 cm^−1^, 883 cm^−1^, 823 cm^−1^, and 702 cm^−1^ and (2) 3304 cm^−1^, 3264 cm^−1^, 1791 cm^−1^, 1597 cm^−1^, 1486 cm^−1^, 1335 cm^−1^, 1303 cm^−1^, 1252 cm^−1^, 1181 cm^−1^, 1150 cm^−1^, 1088 cm^−1^, 1029 cm^−1^, 978 cm^−1^, and 753 cm^−1^, respectively, or in the vicinity of these wavenumber values. The specific vibrations of ferulic and coumaric phenolic acids show vibrations in the IR spectra at (1) 3424 cm^−1^, 1687 cm^−1^, 1629 cm^−1^, 1535 cm^−1^, 1433 cm^−1^, 1382 cm^−1^, 1290 cm^−1^, 1290 cm^−1^, 127 cm^−1^, 125 cm^−1^, 125 cm^−1^, 898 cm^−1^, 851 cm^−1^, and 762 cm^−1^ and (2) 3500 cm^−1^, 1686 cm^−1^, 1542 cm^−1^, 1410 cm^−1^, 1268 cm^−1^, 1105 cm^−1^, 983 cm^−1^, 872 cm^−1^, and 872 cm^−1^, respectively. Curcumin exhibits vibrations in the theoretical IR spectra at 3432 cm^−1^, 3166 cm^−1^, 3088 cm^−1^, 1702 cm^−1^, 1634 cm^−1^, 1608 cm^−1^, 1550 cm^−1^, 1457 cm^−1^, 1391 cm^−1^, 1391 cm^−1^, and 1608 cm^−1^. Also, 966 cm^−1^, 940 cm^−1^, 909 cm^−1^, 836 cm^−1^, 736 cm^−1^, and slight shifts in the vicinity of these wavenumber values are expected.

The specific vibrations of ferulic and coumaric phenolic acids are found with Gaussian simulation in the IR theoretical spectra at (1) 3424 cm^−1^, 1687 cm^−1^, 1629 cm^−1^, 1535 cm^−1^, 1433 cm^−1^, 1382 cm^−1^, 1290 cm^−1^, 1250 cm^−1^, 1098 cm^−1^, 976 cm^−1^, 898 cm^−1^, 851 cm^−1^, and 762 cm^−1^ and (2) 3500 cm^−1^, 1686 cm^−1^, 1542 cm^−1^, 1410 cm^−1^, 1268 cm^−1^, 1105 cm^−1^, 983 cm^−1^, 872 cm^−1^, 729 cm^−1^, respectively, or in their vicinity. Curcumin exhibits vibrations in the theoretical IR spectra at 3432 cm^−1^, 3166 cm^−1^, 3088 cm^−1^, 1702 cm^−1^, 1634 cm^−1^, 1608 cm^−1^, 1550 cm^−1^, 1457 cm^−1^, 1393 cm^−1^, 1044 cm^−1^, 992 cm^−1^, 966 cm^−1^, 940 cm^−1^, 909 cm^−1^, 836 cm^−1^, and 736 cm^−1^. Also, slight shifts in the vicinity of these wavenumber values are expected. Furthermore, analyzing the experimentally obtained spectra using as reference the theoretical spectra of phenolic compounds whose appearance is noticed in the natural as well as in the artificial biocomposites obtained by DPL process, it is observed that all compounds have been transferred from the hemp seed target to the deposition substrate, and their distribution in the thin layers obtained is inhomogeneous, specific to composite materials. In this regard, it can be observed how acid is missing from the spectrum of the HS-DPL/glass (1), HS-DPL/glass (2), HS-DPL/glass (4), and HST-DPL/glass (1) thin film areas due to the absence of the peak at 3500 cm^−1^. The absence of the vibration peak at 3500 cm^−1^ in the spectrum of the hemp seeds/turmeric target (HS-T target) also demonstrates the inhomogeneity of the hemp seed composition specific to composite materials. Also, an increase in the vibrational intensity in the 1700 cm^−1^ range can be observed in the thin film spectra obtained from the hemp seed/turmeric target, especially HST-DPL/glass (2) and HST-DPL/glass (3). This can be attributed to curcumin, including the interaction of curcumin with THC, since the phenomenon of increasing the intensity of the vibration of carbonyl groups in THC is not found in the case of deposits resulting from the ablation of the target from turmeric-free hemp seed (HS-DPL/glass).

**Figure 4:**
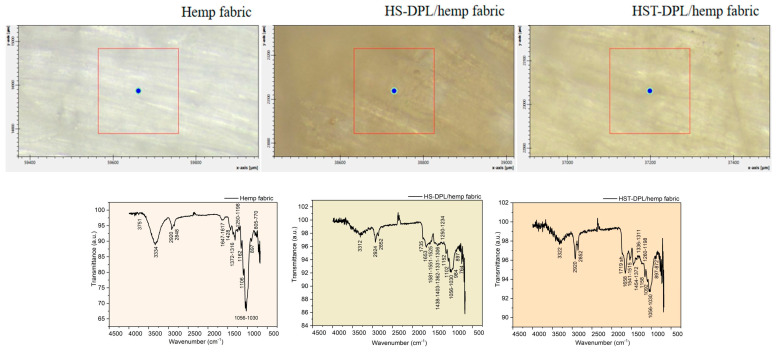
Micro-FTIR ATR spectra of the hemp fabric and of the thin films deposited on the hemp fabric by DPL technique (HS-DPL/hemp fabric and HST-DPL/hemp fabric).

At the same time, there is a slight shift of the peak from 1746 cm^−1^ in the spectra of the HS-target and HST-target targets to 1734 cm^−1^, 1732 cm^−1^, and 1737 cm^−1^ in the thin film spectra (HS-DPL/glass (1)–(4) and HST-DPL/glass (1)–(3)). When depositing on the hemp fabric substrate, when using both HS and HST targets, there is a decrease in the carbonyl vibration in the range of 1700 cm^−1^ and a shift in the spectra obtained by Micro-FTIR analysis performed on the thin layers from 1746 cm^−1^ to 1735 cm^−1^ for HS-DPL/hemp fabric deposition and 1719 cm^−1^ for the HST-DPL/hemp fabric deposition (Figure 4). Bruker Optics published [35] the ATR analysis of the hemp compounds, THC and CBD, and carboxylic acids extracted from cannabis flowers. It is worth noting the presence of vibration in the area of 1700 cm^−1^ in the THC spectrum which we also find in the analyzed samples both for the hemp seeds and for the thin films obtained. Important to notice is that vibration in the 1700 cm^−1^ range does not occur in the CBD [35] spectrum. These results also correspond to the Gaussian 6 simulation of the IR spectra for CBD and THC.

The hemp fabric used as a support for the deposition of thin layers starting from the HS and HST target, after intense and long processing both by mechanical processes of the removal of the wood component and by thermal and chemical processes (boiling with caustic soda and hydrogen peroxide), nevertheless, retains some of the phenolic compounds, which are denoted by the vibrations of the specific phenolic groups of 3334 cm^−1^, 1647 cm^−1^, 1617 cm^−1^, 1428 cm^−1^, 1372 cm^−1^, 1316 cm^−1^, 1250 cm^−1^, 1198 cm^−1^, and 1162 cm^−1^ (Figure 4). During dual pulsed laser deposition, some of the ablated material from the HS or HST target enters the fabric gaps or is absorbed into the fiber, which explains the mitigation of the intensity of some vibration bands compared to those of the targets. These spectra obtained with Micro-FTIR in ATR mode when analyzing thin films deposited on the hemp fabric substrate are actually of the composite manufactured for use as a component of the transdermal drug delivery device.

The phenolic compounds in the thin films deposited on the hemp fabric as HS-DPL/hemp fabric and HST-DPL/hemp fabric are identified by vibrations of their specific functional groups and skeletal vibrations, namely (1) 3312 cm^−1^, 1735 cm^−1^, 1653 cm^−1^, 1581 cm^−1^, 1551 cm^−1^, 1525 cm^−1^, 1438 cm^−1^, 1403 cm^−1^, 1362 cm^−1^, 1331 cm^−1^, 1306 cm^−1^, 1250 cm^−1^, 1234 cm^−1^, 1198 cm^−1^, 1158 cm^−1^, 1102 cm^−1^, 1056 cm^−1^, 1030 cm^−1^, 984 cm^−1^, and 897 cm^−1^ and (2) 3322 cm^−1^, 1719 cm^−1^, 1658 cm^−1^, 1541 cm^−1^, 1515 cm^−1^, 1454 cm^−1^, 1372 cm^−1^, 1336 cm^−1^, 1311 cm^−1^, 1260 cm^−1^, 1152 cm^−1^, 1056 cm^−1^, 1030 cm^−1^, 897 cm^−1^, 872 cm^−1^, and 764 cm^−1^, respectively (Figure 4 and Table S2).

Turmeric added to the hemp seed target to enhance ablation also produced selective ablation intensification effects as shown by the 1732–1734 cm^−1^ carbonyl group vibration band assigned to THC from the HST-DPL/glass (2 and 3) spectra where the transmittance is of 15 a. u. (100 a.u.–85 a. u.) compared to the same peak in the HS-DPL/glass (1, 2, 3, 4) spectra where the transmittance ranges only between 9 a. u. (100 a. u.–91 a. u.) and 6 a. u. (100 a. u.–94 a. u.) (Figure 3). Since the functional groups of the side chains are also found in the spectra of the thin layers at values identical or close to those of the target (Tables S1 and S2), and even if some vibration bands have decreased in intensity or have increased, it means that there have been no structural changes of the chemical components but only different ablation efficiency from one component to another or selective ablation efficiency.

The elemental analysis with EDS technique (Figure S1) shows that including the edge of the stainless steel ring into the DPL process added an insignificant amount of iron to the thin film (only up to 0.55% and an average of 0.29% in weight and maximum 0.13% atomic and an average of 0.07% atomic with the standard deviation ranging for Fe between 13.48% and 32.14%). However, the solution of using turmeric powder to increase ablation yield is more effective for the purpose of the study and subsequent applicability in the medical field.

**Figure 5:**
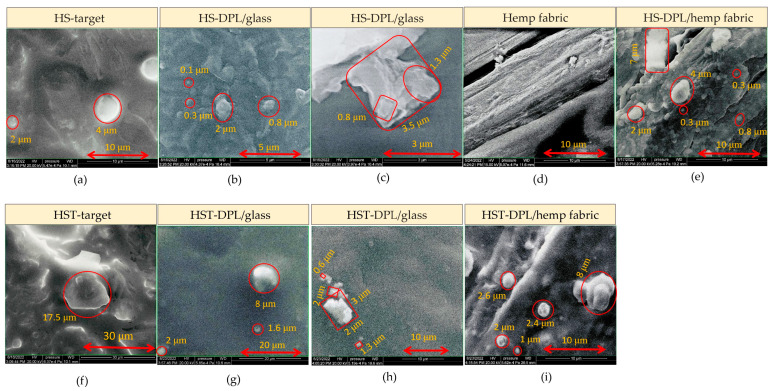
SEM images of the hemp seed target: HS-target (**a**), thin films deposited by DPL technique on glass slab: HS-DPL/glass (**b**,**c**); hemp fabric used as deposition support (**d**) and the thin films obtained with DPL technique on hemp fabric: HS-DPL/hemp fabric (**e**); the target made of hemp seeds mixed with turmeric powder: HST-target (**f**), thin films deposited by DPL technique on glass slab: HST-DPL/glass (**g**,**h**); and the thin films obtained with DPL technique on hemp fabric: HST-DPL/hemp fabric (**i**).

The images acquired with electron microscopy (SEM) highlight the inhomogeneous structure of, the natural biocomposite, hemp seeds and the HS and HST target, with granular formations (Figure 5a and Figure 5f, respectively) that are also found in the artificial biocomposite, i.e., the thin layer obtained by the PLD technique using DPL regime. On the thin-layer SEM images one can see spherical structures of 0.1–2 μm in diameter on the HS-DPL/glass (Figure 5b), 0.3–4 μm on the HS-DPL/hemp fabric (Figure 5d,e), 1.6–8 μm on the HST-DPL/glass (Figure 5g), and 1–8 μm on the HST-DPL/hemp fabric (Figure 5i), as well as angular, apparently amorphous, structures of about 3.5 μm for the HS-DPL/glass (Figure 5c), but also structures with a crystalline appearance of 0.6–3 μm in size for the HST-DPL/glass (Figure 5h). The SEM images thus confirm the morphological similarity of the artificial biocomposite obtained by depositing thin layers with the DPL technique.

### 2.1. Transfer Test

Regarding the substance transfer from the thin layers deposited by the DPL technique on the hemp fabric, tests were carried out on the filter paper.

**Figure 6:**
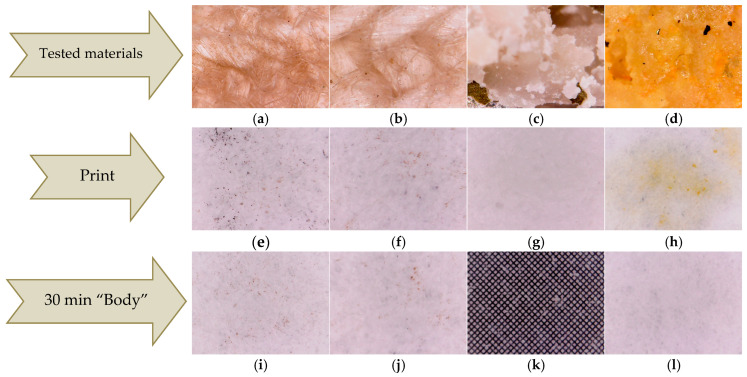
Optical microscopy images of the thin films deposited on hemp fabric: HS-DPL/hemp fabric (**a**) and HST-DPL/hemp fabric (**b**); crushed hemp seeds, HS (**c**); crushed hemp seeds mixed with turmeric powder, HST (**d**); HS-DPL/hemp fabric print test (**e**); HST-DPL/hemp fabric print test (**f**); HS print test (**g**); HST print test (**h**); HS-DPL/hemp fabric transfer test (**i**); HST-DPL/hemp fabric transfer test (**j**); grid 0.1 × 0.1 mm (**k**); and filter paper blank (**l**).

The first test, denoted as print test, consisted of manually pressing the HS-HMP and HST-HMP samples of coated fabrics (Figure 6a,b) on the filter paper. The results were compared with those obtained by pressing crushed hemp seeds (HS) and crushed hemp seeds mixed with turmeric powder (HST) under the same conditions (Figure 6c,d). Pressing the fabric coated the with thin layer, denoted as print tests HS-DPL/hemp fabric and HST-DPL/hemp fabric, led to the deposition of microparticles on the filter paper both in the case of the HS-DPL/hemp fabric and for the HST-DPL/hemp fabric (Figure 6e,f). The print tests performed with HS and HST highlighted a transfer of oils from the hemp seed to the filter paper (Figure 6g, h). The oily stain is very visible for the HST print test (Figure 6h) due to the turmeric color highlighting effect. The print test results show that the HST print consists mainly of hemp oil and turmeric and the HS print consists of hemp oil, while the HS-DPL/hemp fabric prints and the HST-DPL/hemp fabric prints consist of microparticles, the latter two showing no oily stains.

The second test, denoted as the “body” test, consisted in obtaining the transfer of substances as microparticles from the thin layer deposited on the hemp fabric to the filter paper only by keeping it in contact with the filter paper for 30 min at a temperature of 36–37 °C, without pressing. The results of this test showed that the transfer occurred in the form of microparticles in both the HS-DPL/hemp fabric and HST-DPL/hemp fabric samples, as can be seen from Figure 6i,j, with the 0.1 × 0.1 mm gradation in Figure 6k as a reference. This second test looked at whether the transfer of microparticles from the thin layer can take place under the conditions of contact with human skin, i.e., whether the thin layer releases microparticles under the given conditions. Taking into account the print test, it is observed that, from the thin layers deposited from the hemp seed on the hemp fabric, the transfer of microparticles can be achieved under conditions of human body temperature (36–37 °C), and a slight pressing can increase the transfer process. It is also important to note that the microparticles remain independent (do not agglomerate or deform) in the transfer process. The transdermal effect was not tested because the study followed the behavior of the mixture of phenolic compounds in hemp seeds under the action of a 532 nm laser radiation, in the dual pulsed laser (DPL) regime.

If we think of hemp seeds as a biocomposite with a continuous phase mainly of hemp oils and the dispersed phase consisting of various components such as cannabinoids, phenolic acids, and others, the microparticles resulting from the ablation and deposition process should be regarded as microcomposites with most of the constituents of the initial bulk composite. Microcomposites form aggregates but do not mix with each other. They interact only through physical bonds (hydrogen bonds and van der Waals bonds) that hold them together. Transfer tests reflect this aspect of the behavior of microcomposites as individual elements. Each microparticle is thus an element that contains the characteristics of the original biocomposite, i.e., the hemp seed. The DPL process should be seen in this case as the breaking of hemp seeds into small elements suitable for the mass transfer process and possibly for the transdermal process. Microcomposites, with properties and chemical composition of constituents similar to that of the bulk biocomposite material and that aggregate into a compact material that is easy to handle and that can release microparticles over time upon exposure to human body temperature and cross the dermal barrier, represent the concept of a new material designed for TDD systems. These materials can be successfully produced using the pulsed laser deposition technique, either as a single pulse laser (SPL) or as a double pulse laser (DPL) method, the latter being presented in this study for the advantage of being able to work with lower energy lasers. The components are “sealed” in the resulting microcomposites, and their “longevity” when passing through the bloodstream is increased in this way. The components are “sealed” in the resulting microcomposites, and their “longevity” when passing through the bloodstream is increased in this way, unlike products administered orally or even transdermally, but as extracted substances exposed to direct interactions once they enter the body’s organs that can change their chemical profile. To these advantages of the TDD system manufactured by the DPL technique from hemp seeds is added that of the entourage effect due to the content of the microcomposites that constitute the resulting thin layers and which are similar to that of the hemp seed. Practically, we can consider it as a division of the hemp seed into microseeds.

The goal of this work was to develop a new method by which to obtain a new material in the compact form of a thin film but composed of microparticles that can “individuate” and detach under the action of stimuli within a time interval. This objective was achieved by obtaining thin layers using the double pulse laser technique. The thin films produced in this way exhibit the desired morphological structure characteristics, being composed of micro- and sub-microparticles. These micro and nanosized particles that are compacted in a thin film on the deposition support due to physical bonds (hydrogen bonds and van der Waals forces) were detached under the action of external stimuli. The substance transfer tests were performed by pressing, obtaining immediate detachment, and at a temperature similar to the human body when the transfer process was effective within 30 min. This smart behavior of the material makes it suitable for TDD systems.

### 2.2. Antioxidant Activity Test

The antioxidant activity is important to reduce oxidative processes in the biological cells which are harmful for the body [36,37,38]. FTIR analysis highlighted the presence in the thin layers obtained in this study from hemp seeds through the DPL technique of both cannabinoids and phenolic acids, whose antioxidant potential has been demonstrated through studies on various hemp extracts [37]. Since these thin layers obtained through the new method are intended for transdermal transfer, the evaluation of antioxidant activity must be performed directly on the manufactured material as a whole, unlike extraction analysis methods in which each compound is analyzed individually.

**Figure 7:**
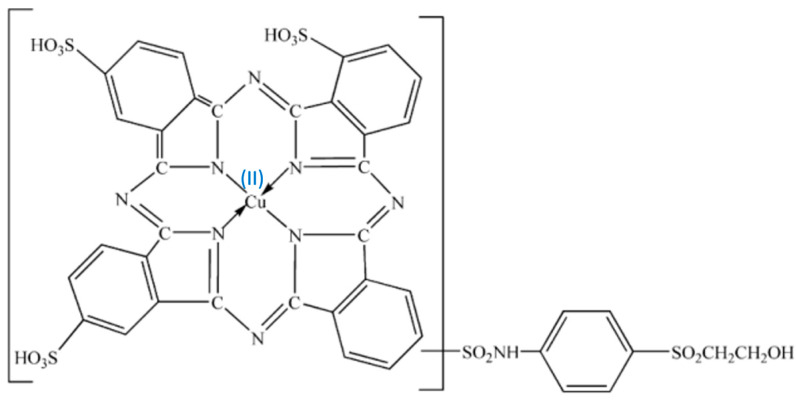
The chemical structure of the dye C.I. Reactive Blue 21.

Among the existing methods, we have adapted the CUPRAC method for the analysis of thin layers containing phenolic compounds. The method is based on the reduction of Cu (II) to Cu (I) by antioxidants. For this purpose we used the dye C.I. Reactive Blue 21 (RB21) which is a copper (II) ion-organic complex, a textile dye with a copper phthalocyanine chromophoric structure onto which the reactive side chain is grafted (Figure 7). In the redox process, the organic complex based on Cu (II) is transformed into an organic complex based on Cu (I). Thus, the oxidation of the phenolic groups of the antioxidants to quinones leads to the reduction of the RB21 and copper (II) ion complex to the RB21 and copper (I) ion complex. The colorimetric evaluation was carried out in the RGB color model using Colorimeter Lab Tools v. 2.25.17. The test was conducted by dripping an aqueous solution of RB21 10 g/l both on the thin film deposited on the hemp fabric (RB21-HS-DPL/hemp fabric) and on that deposited on the glass (RB21-HS-DPL/glass), each being compared with the control samples obtained by dripping the aqueous RB21 solution on the hemp fabric and on the glass substrate and both materials from the control samples being identical to those used as substrates for the thin film deposits. The colorimetric tests were conducted 24 h after treating the samples with the dye solution. The samples were kept in Petri dishes at a room temperature of approximately 24 °C.

**Figure 8:**
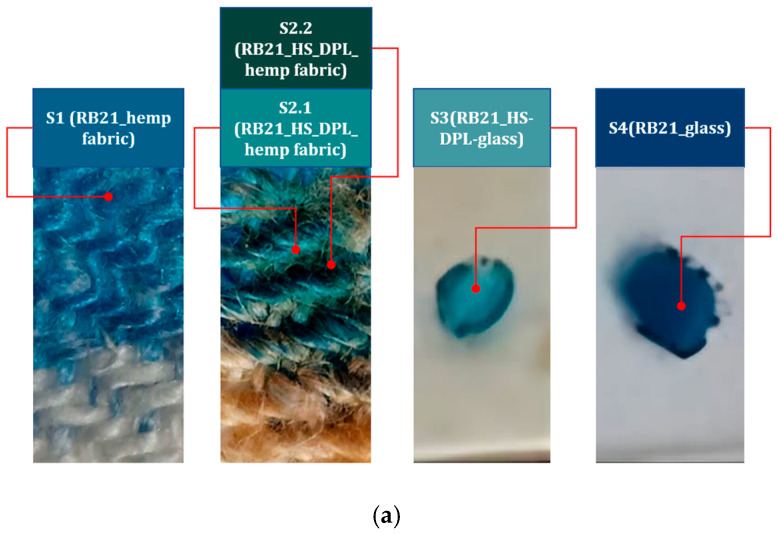

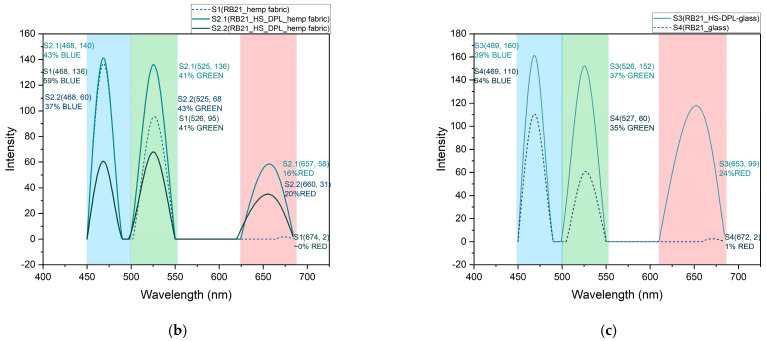
(**a**) The images with the coloristic test; (**b**) The RBG graphic of the colorimetric measurements on the control sample S1 RB21-hemp fabric and on the thin film sample (RB21-HS-DPL/hemp fabric on different areas S2.1. and S2.2) and on the thin film sample on glass S3 (RB21-HS-DPL-glass) compared to the control sample S4 RB21-glass (**c**).

The reduction of Cu (II) ion-complex to Cu (I) ion-complex is studied in the RGB colorimetric model based on the data presented in Table 1 and Table 2. The mitigation in the blue component from 59% for the control sample to 43% and 37% for the two analyzed areas on the RB21-HS-DPL/hemp sample is noticed in the Figure 8a,b, and from 64% for the RB21-glass control sample to 39% for the RB21-HS-DPL/glass sample in the Figure 8a,c. This highlights a change in the ratio between the blue and green components. In this regard, if in the control sample S1 the dominant component is blue of 59% versus 41% green, in the test performed on the thin film deposited on hemp fabric, there is almost an equalization of the percentage between the two components in the analyzed area S2.1 (43% blue versus 41% green) and a decrease in the percentage of the blue component below that of the green component in the analyzed area S2.2 (37% blue versus 43% green). The red component that is almost absent from the control sample S1 is found in the sample resulting from the test performed on the thin film deposited on the hemp fabric both in area S2.1 and in area S2.2 in percentages of 16% and 20%, respectively. The results of the test conducted on the thin film deposited on glass (S3) are more relevant and were compared with the control sample S4 of RB21 dye solution dripped onto the glass substrate without deposition. In this case, interferences due to the substrate were avoided, as the use of hemp fabric as a substrate can lead to interactions with other compounds in hemp fiber, including phenolic compounds, lignin, and as a result of sorption phenomena within the volume of the substrate. In this regard, in Figure 8a, a more uniform coloring is observable in the case of sample S3 compared to S2.1/S2.2. From the graph in Figure 8c, it is observed that there was a decrease in the percentage of the blue component from 64% in the control sample S4 to 39% in the thin film sample S3. At the same time, the ratio between the blue component and the green component changes in favor of the green component in sample S3 (39% blue and 37% green) compared to control sample S4 (64% blue and 35% green). It is also observed, for the thin layer test deposited on glass S3, the red component of 24% which is also almost absent in the test on the control sample on glass S4. The shift towards the green component of the color resulting from the interaction of the RB21 dye aqueous solution with the thin layer deposited from hemp seeds using the DPL technique indicates a process of reduction of Cu (II) ion-complex to Cu (I) ion complex [36] as a result of the interaction with antioxidants represented by cannabinoids, phenolic acids, and other phenolic compounds that were identified by FTIR analysis in the thin films HS-DPL/hemp fabric and HS-DPL/glass. The color change resulted in the test with the RB21 aqueous solution on the control samples and on the samples with thin film deposited by the DPL technique is a bathochromic effect, a shift towards lower wavelength values, which is a characteristic of the reduction of copper from Cu (II) ion-complex to Cu (I) ion complex. The intensity in the RGB model is higher for less color saturation (the highest intensity is for white) and lower for saturated colors (the lowest intensity is for black). The less saturated colors appear to be more specific to Cu(I) ion-complex compared to Cu(II) ion-complex (Figure 8a,c).

### 2.3. Study Limitations and Open Study Perspectives

The study presented focused in particular on the method of obtaining the thin layer of a composition similar to that of hemp seeds.

In order to be able to carry out the application in the manufacture of TDD systems, it will be necessary to continue the study with additional biocompatibility, pharmacokinetic, and bioavailability analyses of the active substances. The biological activity of the deposited material, including the antioxidant activity, requires the development of new methods of analysis or the adaptation of existing ones to the structure and composite specificity of the new material. Clinical trials will also be required.

At this stage it seems difficult to standardize the content of biologically active substances when using hemp seeds resulting from regular crops. In this regard, in order to be able to control the dose of biologically active substances from hemp seeds intended for obtaining smart materials through the laser ablation process, it will be necessary to develop varieties intended for this purpose and to be cultivated in special spaces using specially prepared soils (artificially produced with controlled content), with standardized lighting according to the needs established through specialized studies, as well as by controlling all parameters such as temperature and humidity of the soil and water. Another aspect related to the limitations of the study and the perspectives refers to the adaptation of the methods of quantitative analysis of the active compounds in the thin layers obtained considering their composite nature as well as their interaction with the substrate.

We believe that this idea could become a research topic of interest in the field of biology, pharmacology, physics, chemistry, and agronomy. In this regard, it is even necessary to develop an interdisciplinary project.

## 3. Methods and Materials

**Figure 9:**
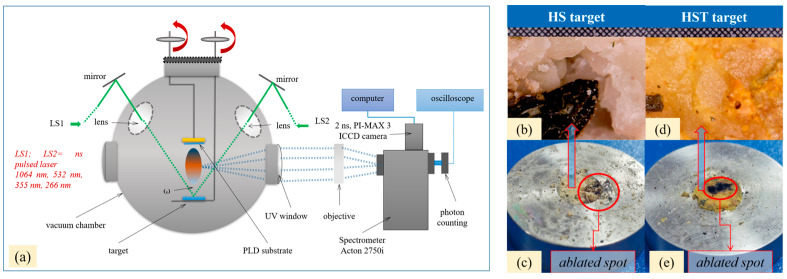
The schematic representation of the installation with the dual pulsed lasers system and deposition chamber (**a**) and the targets used in the dual pulsed laser deposition: optical microscope image with 0.1 × 0.1 mm grid of the hemp seeds: HS target (**b**) and image of the hemp seeds pressed into the stainless steel ring: HS target (**c**); optical microscope image with 0.1 × 0.1 mm grid of the hemp seeds mixed with turmeric powder: HS-T target (**d**) and image of the hemp seeds mixed with turmeric powder pressed into the stainless steel ring: HST-target (**e**).

Dual pulsed laser set-up included the following: The pulsed laser ablation and deposition was conducted using a dual laser system, namely the DPL (dual pulsed laser) regime as per Cocean et al., 2023 [3] and integrated in an installation with a vacuum chamber equipped with a controller for the stepper motor that moves the target. The DPL system consists in the two laser systems Quantel and Q-switched Nd:YAG. (Figure 9a).

A specially designed software provides data to the controller to perform different trajectories and also monitors the time. This way, the target was irradiated simultaneously by two pulsed laser beams each of 30 mJ, a 532 nm wavelength, and a 10 ns pulse width with a 10 Hz repetition rate. The two laser beams were directed so that they formed a 45-degree angle to each other and hit the target at a 45-degree angle of incidence (Figure 1) as was previously presented in the literature as the optimum based on numerical simulation [3]. The deposition time was set to 30 min.

The parameters of the setup, including wavelength, laser exposure time, and the shape and diameter of the focusing spots, were carefully optimized to ensure the formation of a uniform plasma plume. This resulted in a stable particle cloud with consistent direction and shape, without visual alterations to the ablated target. These conditions prevented the uncontrolled piercing of the target by the laser beams, ensuring uniform ablation. The ablated material underwent a cold pressing process in a metal crucible. After conducting several experiments, the energy of the two laser beams was selected to achieve both a uniform plasma and a deposited PLD layer with consistent physicochemical properties, free from impurities caused by potential laser beam reflections or uncontrolled penetration into the crucible walls.

### 3.1. Initial Target

The initial target used in the experiment was made of “organic hulled hemp seeds” (HS-target). The organic hulled hemp seeds are of Lithuanian origin and commercialized, under the brand “Dr. BIO Romania”, and imported and distributed by SC Plafaria SRL, Iasi County, Paun, no. 19, Barnova, Romania. No chemical or physical treatments were used to prepare the hemp seeds before the target was formed, in accordance with the purpose of this research, namely, to reduce the procedures to a minimum and to capitalize on the effects induced by laser radiation.

### 3.2. HS-Target

**Figure 10:**
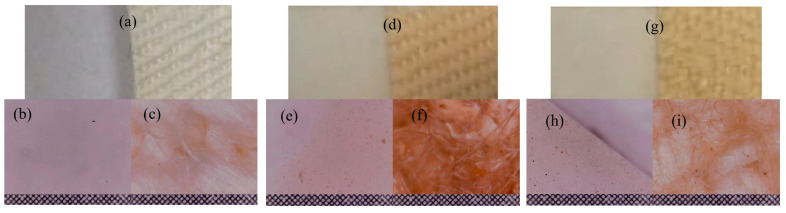
Image of the obtained thin films on the glass and hemp fabric supports under the DPL deposition process applied to the hemp seeds (**a**) and the optical microscope images of the thin films on glass (**b**) and on the hemp fabric as deposition support (**c**); image of the thin film resulted from enhanced ablation and deposition by including the stainless steel ring edge in the DPL process (HS-DPL/glass and HS-DPL/hemp fabric) (**d**) and the optical microscope images with 0.1 × 0.1 grid of the resulted thin films after enhancing the ablation with stainless steel on glass: HS-DPL/glass (**e**) and on the hemp fabric as deposition support: HS-DPL/hemp fabric (**f**); image of the thin film resulted from enhanced ablation and deposition by adding turmeric powder in the hemp seed target (HST-DPL/glass and HST-DPL/hemp fabric) (**g**) and the optical microscope images with the 0.1 × 0.1 grid of the resulted thin films after enhancing the ablation with turmeric on glass: HST-DPL/glass (**h**) and on hemp fabric as deposition support: HST-DPL/hemp fabric (**i**).

The hemp seeds (Figure 9b) were compressed into a stainless steel ring using a hydraulic press under a 100 atm pressure, and the target denoted as HS-target (Figure 9c) was prepared. The pressure of 100 atm was sufficient to compact the seeds in the stainless steel ring with a 12 mm inner diameter, which was used as a mold without affecting the integrity of the seeds. The first DPL deposition from the HS-target resulted in a very thin and barely noticeable layer (Figure 10a–c). In order to improve the deposition yield, the two laser beams were focused on an area also including the edge of the stainless steel ring into which the hemp seed had been pressed. The ablation increased significantly, and the consistency of the deposited layer became evident as can be seen in the Figure 10d–f, and it is in accordance with our previous studies that indicate iron as an enhancing element in the laser ablation process [30].

### 3.3. HS-T Target

A second target was prepared with the aim to improve the ablation using a biocompatible material suitable for the main purpose of the experiment, namely, to obtain a thin layer that can be incorporated into a TDD system. The ablation enhancing material was chosen to be turmeric powder considering the previous results obtained in curcuminoid deposition process reported by Cocean et al., 2021, in which a consistent thin film was obtained without using any additives and a high absorption coefficient of turmeric for the 532 nm wavelength laser beam was proved by the cited experiment [4]. Thus, the second target was made of “organic hulled hemp seeds” mixed with turmeric powder in a ratio of 75% to 25% (Figure 9d). The mixture was also pressed into the stainless steel ring using the same procedure as for the HS-target, resulting in the HST-target (Figure 9e). The turmeric powder used is of Indian origin and commercialized by Sanflora Bucuresti, Romania, of 9.32% humidity and an average granular size of the order of tens of micrometers. The images of the thin films produced from the HST-target by DPL ablation and the deposition process are presented in the Figure 10g–i.

### 3.4. Deposition Substrates

The thin film deposition was performed on glass slab substrates and on hemp fabric substrates (Figure 10). The glass slab was cleaned with ethyl alcohol before deposition. The hemp fabric used as a substrate was produced by SC Hemp Supply, Falticeni, Suceava County, Romania, under the name of Silvana-R, and was of twill texture woven from wet spun yarns of fiber resulted from pool-retted hemp stalks. Before depositing, the fabric was subjected only to a steaming process with an iron to remove any impurities and to improve the adsorption of the material to be deposited as a result of the yarns swelling by steam penetrating through the constituent microfibrils.

The coated materials resulted from the hemp seed target (HS target) by the DPL technique are denoted as HS-DPL/glass when deposited on glass slab and HS-DPL/hemp fabric when deposited on hemp fabric. The coated materials resulted from the target made of hemp seeds mixed with turmeric powder using the DPL technique are denoted as HST-DPL/glass when deposited on glass slab and HST-DPL/hemp fabric when deposited on hemp fabric.

### 3.5. Methods of Analysis

In order to evaluate the chemical composition of the deposition, Micro-FTIR analysis was performed on the thin films obtained on the glass slab support and on hemp fabric and compared to the FTIR spectra obtained for the materials of the HS-target and HST-target. The Micro-FTIR spectra were obtained in the ATR analyzing mode applied directly on the thin films, using Micro-FT-IR LUMOS II, Bruker Optik GmbH, Ettlingen, Germany. The FTIR spectra were obtained using Bomem MB154S spectrometer at an instrumental resolution of 4 cm^−1^ (Bomem, ABB group, Saint-Laurent, QC, Canada), the sample being incorporated in KBr, and a pellet was produced using a 100 atm pressure, the same pressure as used to compact the hemp seeds and hemp seeds with turmeric powder in the stainless steel ring used as targets [4]. For a better evaluation of the vibration bands assigned to the phenolic compounds (cannabinoid and phenolic acids) in the target and thin film spectra, a simulation of IR spectra and molecular structures was conducted with the software GAUSSIAN 6.

The Scanning Electron Microscope coupled with Energy Dispersive X-Ray (SEM-EDS) investigation, performed with Vega Tescan LMH II, Brno, Czech, provided information on the morphology and elemental composition of the studied thin film materials compared to the targets and the hemp fabric used as deposition support. For energy dispersive spectroscopy a SE detector was used at a 30 kV filament supply and a working distance of 15.5 mm and, for EDX, the Bruker detector X-Flash 6/30 with automatic mode detection and precise experiment. For electron microscopy investigations, the samples were metallized with Gold (7 nm) using a Luxor deposition system dedicated to weak or non-conductive samples. The tests were performed in high vacuum mode (3–6 × 10^−4^ Pa) with a current absorbed on the sample of 0.1 microA. Also, optical microscopy (OM) was performed using a Namicon ISMM1000 trinocular inverted metallographic microscope (INSIZE, Suzhou, China), equipped with a MotiCam camera (10+, 10.0 MP), specialized in microscopic analysis, and Motic Images Plus 3.0 (×86) software, version 3.0.12.41 (Motic, China-Group Co., Ltd. 2015, Hong Kong, China).

## 4. Conclusions

Hemp seeds are source of compounds with great potential for the pharmaceutical industry. The micro- and nanoparticle structure of the thin layers obtained by the laser ablation and deposition on biocompatible textiles gives the advantage of easy handling, as well as more effective control over the use of substances with a risk of intoxication and severe side effects. Starting from the idea of using the plant or a part of the plant (stem, leaves, flowers, fruits, root, etc.) as a whole (whole plant medicine concept) to benefit from the combinatorial effect between the various cannabinoids, as well as with other active compounds from hemp seeds (entourage effect), laser ablation with the deposition of thin layers deserves special attention in the attempt to obtain materials for transdermal drug delivery (TDD) systems. Thus, some of the negative effects of Tetrahydrocannabinol (THC) would be reduced by interacting with the cannabidiol (CBD) component, and people can benefit from its appetite-increasing effects in anorexia and as an antiemetic, as well as, a sleep apnea reliever. The obtaining of thin layers consisting of micro- and nanogranular structures as resulted from electron microscopy (SEM), also evidenced by microparticle transfer tests on filter paper, shows their availability for transdermal transfer. In addition, the microparticles (some being aggregates of smaller particles) are actually microcomposites as shown by the ATR analysis performed with the Micro-FTIR technique, so that a mixture of compounds would be delivered through the skin with each microparticle transferred from the thin layer under the action of human body temperature without requiring any additional devices. The test with the aqueous solution of the dye RB21, analyzed colorimetrically, was adapted based on the CUPRAC test for analyzing the antioxidant potential of reducing Cu(II) ion-complex to Cu(I) ion-complex through colorimetric measurements. The test has been adapted for thin films and can be further developed for this type of analysis.

Although the study is at the beginning of its journey and further investigations and adjustments are needed, including clinical trials, the results are encouraging and promise that the new technique for producing TDD systems will find applications in the pharmaceutical industry. The method can be extended to the realization of artificial biocomposites starting from other natural biocomposites used as ablation targets, as well as for the manufacture of artificial biocomposites by depositing thin layers with the DPL (dual pulsed laser) or SPL (single pulsed laser) technique, by ablating targets produced according to a mimetic model from components that exhibit entourage effect behavior. Another future application of the method could be the production of transdermal patches for the delivery of nutrients to patients who require artificial feeding. Ongoing research may further elucidate their therapeutic potential and new techniques to fabricate transdermal drug delivery systems (TDD systems), paving the way for novel approaches in health and wellness.

## Figures and Tables

**Table 1:** RGB data obtained in the colorimetric analysis for the antioxidant test on HS-DPL-hemp fabric.

Sample	R	G	B	R %	G%	B%
S1 (RB21-hemp fabric)	0	95	136	0	41	59
S2.1 (RB21-HS-DPL-hemp fabric)	58	136	140	16	41	43
S2.2 (RB21-HS-DPL-hemp fabric)	31	68	60	20	43	37

**Table 2:** RGB data obtained in the colorimetric analysis for antioxidant test on HS-DPL-glass.

Sample	R	G	B	R %	G%	B%
S3 (RB21-DPL-glass)	99	152	160	24	37	39
S4 (RB21-glass)	2	60	110	1	35	64

## Data Availability

The original contributions presented in this study are included in the article/Supplementary Materials. Further inquiries can be directed to the corresponding authors.

## Supplementary Materials

The following supporting information can be downloaded at https://www.mdpi.com/article/10.3390/ijms26167852/s1.
