# Supplementary material for: Laser-Induced Ablation of Hemp Seed-Derived Biomaterials for Transdermal Drug Delivery
Source: Int J Mol Sci. 2025 Aug 14;26(16):7852. doi: 10.3390/ijms26167852 (PMC12386652; doi:10.3390/ijms26167852)
Supplement: Supplementary file 1 [file ijms-26-07852-s001.zip › ijms-3759099-supplementary.pdf]

Journal: IJMS (ISSN 1422-0067)
Manuscript ID: ijms-3759099
Type: Article
Title: Laser-Induced Ablation of Hemp Seed-Derived Biomaterials for Transdermal Drug Delivery
Authors: Alexandru Cocean , Georgiana Cocean , Silvia Garofalide , Nicanor Cimpoesu , Daniel Alexa , Iuliana Cocean * , Silviu Gurlui *
Section: Molecular Pathology, Diagnostics, and Therapeutics
Topical Collection: Feature Papers in Molecular Pathology, Diagnostics, and Therapeutics

---

Annex 1

Table S1 (supplementary). Vibration bands and assigned functional groups in the FTIR and micro-FTIR spectra of the target consisting in hemp seeds and hemp seeds mixed with turmeric powder and of the different areas on the thin films deposited on the glass slab; based on the data in Figure 3.

**Vibration bands [cm^{-1}]**											**Functional groups identified based on Pretsch et al., 2009 [34] and on the Gaussian 6 IR spectra simulation performed in this study (Figure 2)**
**HS-target**	**HS-target ablated**	**HS-DPL/ glass (1)**	**HS-DPL/ glass (2)**	**HS-DPL/ glass (3)**	**HS-DPL/ glass (4)**	**HS-T-target**	**HST-target ablated**	**HST-DPL/ glass (1)**	**HST-DPL/ glass (2)**	**HST-DPL/ glass (3)**	
-	-	-	3829	-	-	3855<br>3748	-<br>3741	-	-<br>3749	-	OH stretching, free
3554<br>3290	3554<br>3274	3495<br>3375<br>3289	3460<br>3343<br>3289	3558<br>3375<br>3289	3410<br>3312<br>3258	3411<br>3292	-<br>3313	3472<br>3319	3524<br>3352	3518<br>3335	OH and NH stretching, free and H-bonded<br>C-H aromatic stretching (3200-3312 cm^{-1} bands are assigned to aromatic CH and phenolic OH)<br>Lignanamides (3364 cm^{-1})<br>Gaussian simulation: CBD (3313 cm^{-1}; 3161 cm^{-1}); THC (3304 cm^{-1}; 3264 cm^{-1}); ferulic acid (3424 cm^{-1}); coumaric acid (3500 cm^{-1}); curcumin (3432 cm^{-1})
3011	3011	-	-	-	-	3006	3007	-	-	-	CH aromatic stretching<br>Gaussian simulation: CBD (3119 cm^{-1}; 3059 cm^{-1}; 3018 cm^{-1}); curcumin (3166 cm^{-1}; 3088 cm^{-1})
2928	2928	2934	2934	2934	2934	2924	2924	2932	2920	2934	CH aliphatic asymmetric stretching
2854	2854	2857	2857	2857	2857	2848	2848	2857	2849	2859	CH aliphatic symmetric stretching
2354	2354	-	-	-	-	2374	2374	-	-	-	CO_2
-	-	-	-	-	-	1866	1866	-	-	-	C=O stretching (assigned to hemp oil)<br>Esthers; aldehyde ether
1746	1746	1734	1734	1734	1734	1746	1746	1732	1732	1737	C=O stretching (assigned to THC; hemp oil; curcumin)<br>Esthers; cyclopentanone (1745 cm^{-1}); aldehyde ether<br>Gaussian simulation: THC (1791cm^{-1}); curcumin (1702 cm^{-1})
1648	1648	1648	1636	1648	1661	1651	1651	1657	1657	1661	C=O stretching in amides<br>Lignanamides (1656 cm^{-1})<br>C=C bending in alkenes (specific to cannabinoids side chain)<br>Gaussian simulation: CBD (1617 cm^{-1}); ferulic acid (1687 cm^{-1}; 1629 cm^{-1}); coumaric acid (1686 cm^{-1}); curcumin (1634 cm^{-1}; 1608 cm^{-1})
1540	1540	1552	1552	1552	1541	1532	1532	1528	1528	1530	NH bending (deformation) in amides<br>Lignanamides (1514 cm^{-1})<br>C=C bending in alkenes (specific to CBD side chain)<br>Gaussian simulation: THC (1597 cm^{-1}); ferulic acid (1535 cm^{-1}); coumaric acid (1542 cm^{-1}); curcumin (1550 cm^{-1})
1460	1460	1453	1453	1453	1466	1467	1457	1462	1462	1451	OH bending in alcohols and COOH (carboxylic acids)<br>CH bending of methyl and methylene group<br>Gaussian simulation: CBD (1473 cm^{-1}); THC (1486 cm^{-1}); ferulic acid (1433 cm^{-1}); coumaric acid (1410 cm^{-1}); curcumin (1457 cm^{-1})
1394<br>1312	1394<br>1312	1378	1378	1378	1358	1392	1381	1376	1376	1379	OH bending in phenols<br>CH_3 bending

											Gaussian simulation: CBD (1392 cm^{-1}); THC (1335 cm^{-1}; 1303 cm^{-1}); ferulic acid (1382 cm^{-1}); curcumin (1393 cm^{-1})
1246	1246	1237sh	1237sh	-	1250	1230	1241sh	1246sh	1235sh	1239sh	Ar-C-OH bending<br>CN stretching in aromatic amine<br>CO ring skeletal stretching in epoxides<br>Gaussian simulation: CBD (1290 cm^{-1}; 1210 cm^{-1}); THC (1252 cm^{-1}); ferulic acid (1290 cm^{-1}; 1250 cm^{-1}); coumaric acid (1268 cm^{-1})
1162	1162	1162sh	1162sh	1162sh	1162sh	1158	1158	1160	1148	1164	Ar-C-OH bending<br>C=C bending in alkenes<br>CO ring skeletal vibrations in epoxides.<br>Gaussian simulation: THC (1181 cm^{-1}; 1150 cm^{-1}); curcumin (1128 cm^{-1})
1098sh	1098sh	-	-	-	1023	1092sh	1090sh	-	1040	1098	C=C bending in alkenes<br>Gaussian simulation: CBD (1036 cm^{-1}); THC (1088 cm^{-1}; 1029 cm^{-1}); ferulic acid (1098 cm^{-1}); coumaric acid (1105 cm^{-1}); curcumin (1044 cm^{-1})
1000sh<br>908	1000sh	965<br>928	962<br>928	928	-	984sh	992sh	988-<br>901	976-<br>901	980-<br>915	C=C bending in alkenes<br>Gaussian simulation: CBD (966 cm^{-1}; 934 cm^{-1}); THC (978 cm^{-1}); ferulic acid (976 cm^{-1}); coumaric acid (983 cm^{-1}); curcumin (992 cm^{-1}; 966 cm^{-1}; 940 cm^{-1}; 909 cm^{-1})
851	851	-	-	-	894						C=C bending in alkenes<br>Gaussian simulation: CBD (883 cm^{-1}; 823 cm^{-1}); ferulic acid (898 cm^{-1}; 851 cm^{-1}); coumaric acid (872 cm^{-1}); curcumin (836 cm^{-1})
714	714	774	761	761	732	715	715	772	749	732	C=C skeletal vibrations<br>Gaussian simulation: CBD (702 cm^{-1}); THC (753 cm^{-1}); ferulic acid (762 cm^{-1}); coumaric acid (729 cm^{-1}); curcumin (736 cm^{-1})

Table S2 supplementary. Vibration bands and assigned functional groups in the FTIR and micro-FTIR spectra of the target consisting in hemp seeds and hemp seeds mixed with turmeric powder and of the different areas on the thin films deposited on the hemp fabric support; based on the data in Figure 2.

**Vibration bands [cm^{-1}]**					**Functional groups identified based on Pretsch et al., 2009 [34] and on the Gaussian 6 IR spectra simulation performed in this study (Figure 2)**
**HS-target**	**HST-target**	**Hemp fabric**	**HS-DPL/hemp fabric**	**HST-DPL/hemp faric**	
-	3855	-	-	-	OH stretching, free
	3748	3751	-	-	OH stretching, free
3554-3290	3411-3292	3334	3312	3322	OH and NH stretching, free and H-bonded<br>CH aromatic stretching (3200-3312 cm^{-1} bands are assigned to aromatic CH and phenolic OH)<br>Lignanamides (3364 cm^{-1})<br>Gaussian simulation: CBD (3313 cm^{-1}; 3161 cm^{-1}); THC (3304 cm^{-1}; 3264 cm^{-1}); Ferulic Acid (3424 cm^{-1}); Coumaric Acid (3500 cm^{-1}); Curcumin (3432 cm^{-1})
3011	3065sh<br>3006	-	-	-	CH aromatic stretching<br>Gaussian simulation: CBD (3119 cm^{-1}; 3059 cm^{-1}; 3018 cm^{-1}); Curcumin (3166 cm^{-1}; 3088 cm^{-1})
2928	2924	2920	2924	2920	CH aliphatic asymmetric stretching
2854	2848	2848	2852	2852	CH aliphatic symmetric stretching
2354	2374	-	-	-	CO_2
-	1866	-	-	-	C=O stretching (assigned to hemp oil [ ])<br>Esthers; aldehyde ether

1746	1746	-	1735	1719sh	C=O stretching (assigned to THC, hemp oil , curcumin)<br>Esthers; cyclopentanone (1745 cm^{-1}); aldehyde ether<br>Gaussian simulation: THC (1791 cm^{-1}); Curcumin (1702)
1648	1651	1647-1617	1653	1658	C=O stretching in amides<br>Lignanamides (1656 cm^{-1})<br>C=C bending in alkene (specific to CBD side chain<br>Gaussian simulation: CBD (1617cm^{-1}); Ferulic Acid (1687 cm^{-1}; 1629 cm^{-1}); Coumaric Acid (1686 cm^{-1}); Curcumin (1634 cm^{-1}; 1608 cm^{-1})
1540	1532	-	1581<br>1551<br>1525	1541<br>1515	NH bending (deformation) in amides<br>Lignanamides (1514 cm^{-1})<br>C=C bending in alkene (specific to CBD side chain)<br>Gaussian simulation: THC (1597 cm^{-1}); Ferulic Acid (1535 cm^{-1}); Coumaric Acid (1542 cm^{-1}); Curcumin (1550 cm^{-1})
1460	1467	1428	1438<br>1403	1454	OH bending in alcohols and COOH (carboxylic acids)<br>CH bending of methyl and methylene group<br>Gaussian simulation: CBD (1473 cm^{-1}); THC (1486 cm^{-1}); Ferulic Acid (1433 cm^{-1}); Coumaric Acid (1410 cm^{-1}); Curcumin (1457 cm^{-1})
1394<br>1312	1392	1372<br>1316	1362<br>1331<br>1306	1372<br>1336<br>1311	OH bending in phenols<br>CH_3 bending<br>Gaussian simulation: CBD (1392 cm^{-1}); THC (1335 cm^{-1}; 1303 cm^{-1}); Ferulic Acid (1382 cm^{-1}); Curcumin (1393 cm^{-1})
1246	1230	1250	1250<br>1234	1260	Ar-C-OH bending<br>CN stretching in aromatic amine<br>CO ring skeletal vibrations in epoxides<br>Gaussian simulation: CBD (1290 cm^{-1}; 1210 cm^{-1}); THC (1252 cm^{-1}); Ferulic Acid (1290 cm^{-1}; 1250 cm^{-1}); Coumaric Acid (1268 cm^{-1})
1162	1158	1198;1162	1198<br>1158	1152	Ar-C-OH bending<br>C=C bending in alkenes<br>CO ring skeletal vibrations in epoxides<br>Gaussian simulation: THC (1181 cm^{-1}; 1150 cm^{-1})
1098sh	1092sh	1106	1102<br>1056<br>1030	1056<br>1030	C=C bending in alkenes<br>Gaussian simulation: CBD (1036 cm^{-1}); THC (1088 cm^{-1}; 1029 cm^{-1}); Ferulic Acid (1098 cm^{-1}); Coumaric Acid (1105 cm^{-1}); Curcumin (1044 v)
1000sh<br>908	984sh	-	984	-	C=C bending in alkenes

					Gaussian simulation: CBD (966 cm^{-1}; 934 v); THC (978 cm^{-1}); Ferulic Acid 976 cm^{-1}); Coumaric Acid (983 cm^{-1}); Curcumin (992 cm^{-1}; 966 cm^{-1}; 940 cm^{-1}; 909 cm^{-1})
851	-	897<br>805	897	897<br>872	C=C bending in alkenes<br>Gaussian simulation: CBD (883 cm^{-1}; 823 cm^{-1}); Ferulic Acid (898 cm^{-1}; 851 cm^{-1}); Coumaric Acid (972 cm^{-1}); Curcumin (836 cm^{-1})
714	715	770	-	764	CC skeletal vibrations<br>Gaussian simulation: CBD (702 cm^{-1}); THC (753 cm^{-1}); Ferulic Acid (762 cm^{-1}); Coumaric Acid (729 cm^{-1}); Curcumin (736)

Samples		HS target area1	HS target area2	Average HS target	HS DPL/ glass area1	HS DPL/ glass area2	HS DPL/ hemp fabric area1	HS DPL/ hemp fabric area2	Average HS DPL	HST target area1	HST target area2	Average HST target	HST DPL/ glass area1	HST DPL/ glass area2	HST DPL/ glass area3	HST DPL/ hemp fabric area1	HST DPL/ hemp fabric area2	HST DPL/ hemp fabric area3	Average HST DPL
Elements – Weight %	C	76.75	78.48	77.62	72.27	36.17	53.12	55.87	54.36	77.93	73.32	75.63	52.53	11.54	70.14	63.26	68.62	71.52	56.27
	O	23.20	21.52	22.36	20.95	50.01	46.16	43.49	40.15	21.12	26.39	23.76	29.30	57.54	19.75	36.74	31.38	28.48	33.87
	Na	-	-	-	0.94	7.51	-	-	2.11	-	-	-	7.01	14.78	4.13	-	-	-	4.32
	Ca	-	-	-	0.14	0.21	0.30	-	0.16	-	-	-	0.61	0.44	0.36	-	-	-	0.24
	Mg	-	-	-	0.35	1.18	-	-	0.38	-	-	-	1.44	3.01	0.79	-	-	-	0.87
	Al	-	-	-	0.18	0.12	-	-	0.08	0.33	-	0.17	0.30	-	0.19	-	-	-	0.08
	Si	-	-	-	1.42	4.74	0.08	-	1.56	-	-	-	8.50	12.69	4.53	-	-	-	4.29
	Fe	-	-	-	0.55	-	0.20	0.40	0.29	-	-	-	-	-	-	-	-	-	-
	K	-	-	-	0.09	0.06	-	-	0.04	0.39	0.29	0.34	-	-	0.11	-	-	-	0.02
	S	0.06	-	0.03	0.11	-	-	-	0.03	0.06	-	0.03	-	-	-	-	-	-	-
	Cl	-	-	-	0.09	-	-	-	0.02	-	-	-	-	-	-	-	-	-	-
	Ni	-	2.64	1.32	-	-	0.11	0.24	0.09	-	-	-	-	-	-	-	-	-	-
	P	-	-	-	0.12	-	0.03	-	0.04	0.17	-	0.09	-	-	-	-	-	-	-
	Cr	-	-	-	0.15	-	-	-	0.04	-	-	-	-	-	-	-	-	-	-
	Sn		-	-	-	-	-	-	-	-	-	-	0.30	-	-	-	-	-	0.05
Elements – Atomic %	C	81.49	82.93	82.21	80.10	45.00	60.38	63.02	62.13	82.78	78.65	80.72	63.38	16.61	78.21	69.64	74.44	76.99	63.21
	O	18.49	17.07	17.78	17.43	46.71	39.39	36.83	35.09	16.84	21.25	19.05	26.54	62.15	16.53	30.36	25.56	23.01	30.69
	Na	-	-	-	0.54	4.88	-	-	1.36	-	-	-	4.42	11.11	2.41	-	-	-	2.99
	Ca	-	-	-	0.05	0.08	0.10	-	0.06	-	-	-	0.22	0.19	0.12	-	-	-	0.09
	Mg	-	-	-	0.19	0.72	-	-	0.23	-	-	-	0.86	2.14	0.43	-	-	-	0.57
	Al	-	-	-	0.09	0.07	-	-	0.04	0.16	-	0.08	0.16	-	0.10	-	-	-	0.04
	Si	-	-	-	0.68	2.52	0.04	-	0.81	-	-	-	4.38	7.81	2.16	-	-	-	2.39
	Fe	-	-	-	0.13	-	0.05	0.10	0.07	-	-	-	-	-	-	-	-	-	-
	K	-	-	-	0.03	0.02	-	-	0.01	0.13	0.10	0.12	-	-	0.04	-	-	-	0.01
	S	0.02	-	0.01	0.04	-	-	-	0.01	0.02	-	0.01	-	-	-	-	-	-	-
	Cl	-	-	-	0.03	-	-	-	0.01	-	-	-	-	-	-	-	-	-	-
	Ni	-	-	-	0.60	-	0.02	0.06	0.17	-	-	-	-	-	-	-	-	-	-
	P	-	-	-	0.05	-	0.02		0.02	0.07	-	0.04	-	-	-	-	-	-	-
	Cr	-	-	-	0.04	-	-	-	0.01	-	-	-	-	-	-	-	-	-	-
	Sn	-	-	-	-	-	-	-	-	-	-	-	0.04	-	-	-	-	-	0.01

(a)

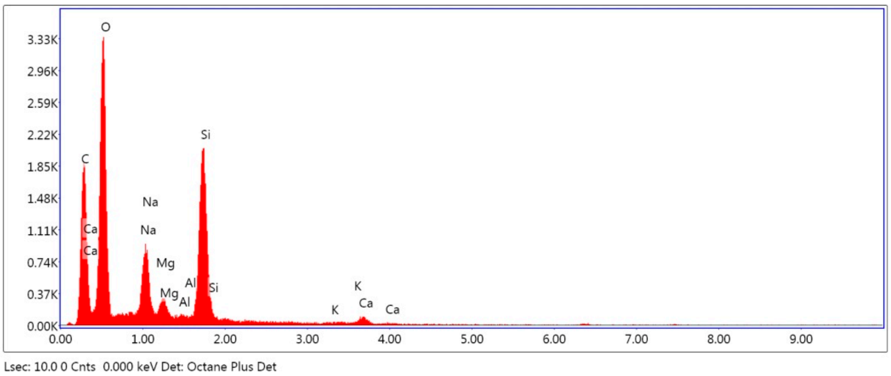

(b)

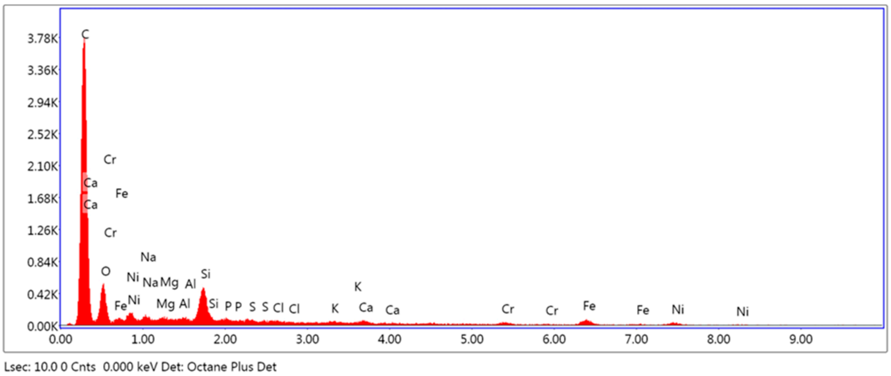

(c)

Figure 1 supplementary. Results of target and thin-film elemental analysis with Energy Dispersive X-Ray (EDS) technique (a) and the spectra of the HS-DPL/glass: analyzed area 1 with no content of iron (b) and analyzed area 2 with the highest content of iron (0.55% weight Fe out of total elements).

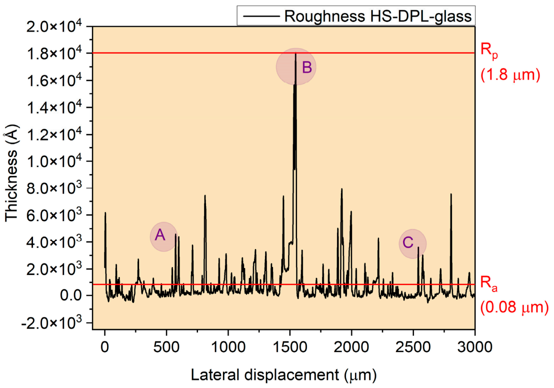
Roughness HS-DPL-glass
Thickness (Å)
Lateral displacement (µm)
R_p (1.8 µm)
R_a (0.08 µm)
A
B
C
2.0×10^4
1.8×10^4
1.6×10^4
1.4×10^4
1.2×10^4
1.0×10^4
8.0×10^3
6.0×10^3
4.0×10^3
2.0×10^3
0.0
-2.0×10^3
0
500
1000
1500
2000
2500
3000

(a)

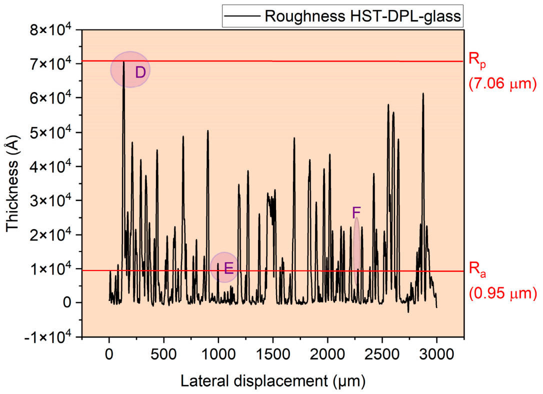
Roughness HST-DPL-glass
Thickness (Å)
Lateral displacement (µm)
R_p (7.06 µm)
R_a (0.95 µm)
D
E
F
8×10^4
7×10^4
6×10^4
5×10^4
4×10^4
3×10^4
2×10^4
1×10^4
0
-1×10^4
0
500
1000
1500
2000
2500
3000

(b)

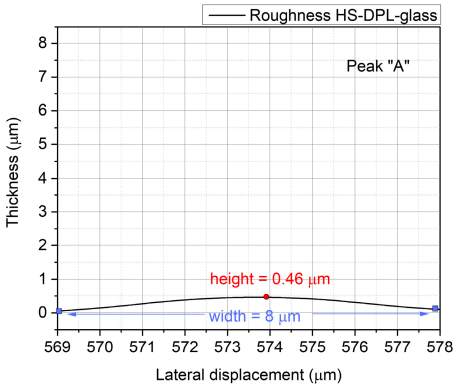
Roughness HS-DPL-glass
Peak "A"
height = 0.46 µm
width = 8 µm
Thickness (µm)
Lateral displacement (µm)
8
7
6
5
4
3
2
1
0
569
570
571
572
573
574
575
576
577
578

(c)

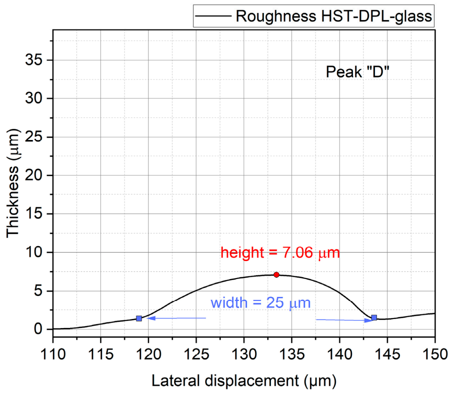
Roughness HST-DPL-glass
Peak "D"
height = 7.06 µm
width = 25 µm
Thickness (µm)
Lateral displacement (µm)
35
30
25
20
15
10
5
0
110
115
120
125
130
135
140
145
150

(d)

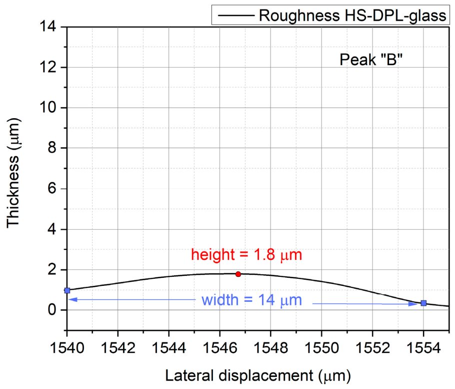
Roughness HS-DPL-glass
Peak "B"
height = 1.8 µm
width = 14 µm
Thickness (µm)
Lateral displacement (µm)
14
12
10
8
6
4
2
0
1540
1542
1544
1546
1548
1550
1552
1554

(e)

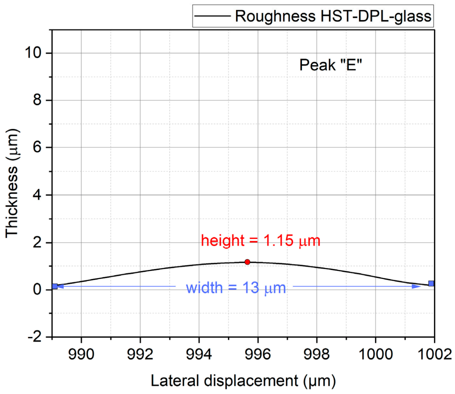
Roughness HST-DPL-glass
Peak "E"
height = 1.15 µm
width = 13 µm
Thickness (µm)
Lateral displacement (µm)
10
8
6
4
2
0
-2
990
992
994
996
998
1000
1002

(f)

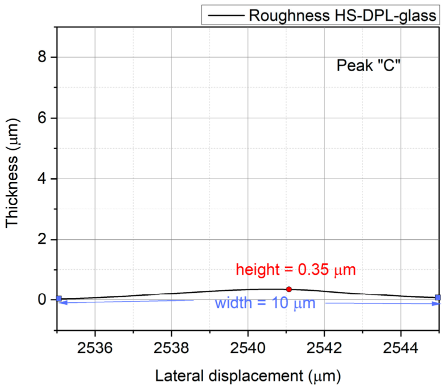

(g)

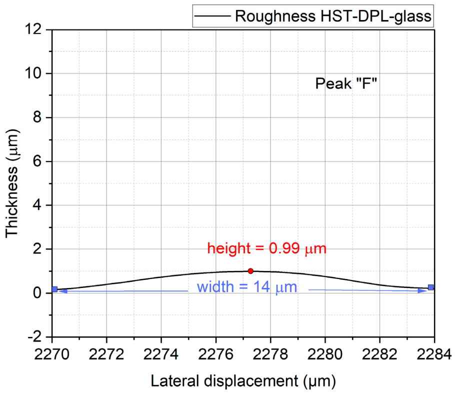

(h)

Figure 2 supplementary. Roughness of the thin films obtained by DPL deposition technique applied on the hemp-seed target: HS-DPL total profile (a) and details on HS-DPL peak “A” (c), HS-DPL peak “B” (e), and HS-DPL peak “C” (g) and HST-DPL total profile (b) and details on HST-DPL peak “D” (d), HST-DPL peak “E” (f), and HST-DPL peak “F”(h).

Table 3 supplementary. The width/height ratios of the detailed HS-DPL peaks A, B, and C and HST-DPL peaks D, E, and F.

Sample	Peak	Width/Height	Ratio	Comments
HS-DPL	A	8 μm / 0.46 μm	17	The ratio of the width/height denotes flat structures that make up the roughness of the thin layer and that are associated with the deposition particles. The explanation lies in the fact that, due to the high energy with which the particles come into contact with the surface on which they are deposited, they are most often significantly flattened. The phenomenon is due to the Plateau–Rayleigh, Rayleigh–Taylor, and Richtmyer–Meshkov instabilities that manifest in the ablation plume on its way from the target to the deposition substrate [33].
	B	14 μm / 1.8 μm	7.7	
	C	10 μm / 0.35 μm	28	
HST-DPL	D	25 μm / 7.06 μm	3	
	E	13 μm / 1.15 μm	11	
	F	14 μm / 0.99 μm	14	

Table 4 supplementary. Roughness parameters of the thin films HS-DPL and HST-DPL deposited on the glass slab. The parameters were calculated using the Gaussian-filter-type long wavelength pass and the cut-off length of $\lambda_c = 3$ mm.

Sample	R_a (μm)	R_p (μm)	R_v (μm)	R_z (μm)	R_q (μm)	R_{sk}	R_{ku}	Comments
	Arithmetical Mean Height	Maximum Peak Height	Maximum Pit Height	Maximum Height	Root Mean Square Height	Skewness	Kurtosis	
HS-DPL	0.08	1.8	0.05	1.85	0.18	4.96	34.04	Rsk > 0 indicates a right skew or positively skewed distribution. It means that the tail on the right side (the larger values) is longer than the tail on the left side (the smaller values). The most of the peaks are concentrated on the left side (the smaller values), and some extreme peaks are on the right side (the larger values). Rku > 3 shows high kurtosis specific to leptokurtic distribution characterized by heavier tails, with much
HST-DPL	0.95	7.06	0.27	7.33	1.62	2.32	6.30	

								more extreme values or outliers for sample HS-DPL. Ra and Rq values denote that the roughness distribution for the HST-DPL sample is more uniform than that of the HS-DPL sample.

Correlating the roughness results with the SEM analysis, it can be stated that the roughness of the thin films is due to the granular structure of the obtained thin films. The granular deposition gives the thin layer a porosity that makes it suitable for the absorption and adsorption of active substances in liquid or gaseous states that could be incorporated for the manufacture of transdermal drug delivery devices. Another advantage of thin layers with micro- and nanogranular structures is that, under certain conditions of temperature and/or pressure, these structures can detach from the layer and cross the dermal barrier with which they are in contact.

The roughness of the thin films was evaluated using the DektakXT Stylus Profilometer (Bruker, Bruker Nano Surfaces Division, 3400 East Britannia Drive, Suite 150, Tucson, AZ 85706). The profile hills and valleys were generated in a standard scan performed on a 3 cm length of the sample surface with the scan resolution of 0.333259 μm, stylus force of 10 mg, and scan duration of 30 s. The roughness parameters were analyzed with the software OriginLab, version 2022, using the application Surface Roughness Parameters.
